# Inhibitors of the Ubiquitin-Mediated Signaling Pathway Exhibit Broad-Spectrum Antiviral Activities against New World Alphaviruses

**DOI:** 10.3390/v15030655

**Published:** 2023-02-28

**Authors:** Niloufar A. Boghdeh, Brittany McGraw, Michael D. Barrera, Carol Anderson, Haseebullah Baha, Kenneth H. Risner, Ifedayo V. Ogungbe, Farhang Alem, Aarthi Narayanan

**Affiliations:** 1 Biomedical Research Laboratory, George Mason University, Manassas, VA 20110, USA; 2 School of Systems Biology, College of Science, George Mason University, Manassas, VA 20110, USA; 3 Department of Chemistry, Physics and Atmospheric Sciences, Jackson State University, Jackson, MS 39217, USA; 4 Department of Biology, College of Science, George Mason University, Fairfax, VA 22030, USA

**Keywords:** alphavirus, Venezuelan Equine Encephalitis Virus, Eastern Equine Encephalitis Virus, ubiquitin proteasome system, ubiquitin signaling pathways, omaveloxolone, bardoxolone methyl, antiviral, antiinflammatory, therapeutics

## Abstract

New World alphaviruses including Venezuelan Equine Encephalitis Virus (VEEV) and Eastern Equine Encephalitis Virus (EEEV) are mosquito-transmitted viruses that cause disease in humans and equines. There are currently no FDA-approved therapeutics or vaccines to treat or prevent exposure-associated encephalitic disease. The ubiquitin proteasome system (UPS)-associated signaling events are known to play an important role in the establishment of a productive infection for several acutely infectious viruses. The critical engagement of the UPS-associated signaling mechanisms by many viruses as host–pathogen interaction hubs led us to hypothesize that small molecule inhibitors that interfere with these signaling pathways will exert broad-spectrum inhibitory activity against alphaviruses. We queried eight inhibitors of the UPS signaling pathway for antiviral outcomes against VEEV. Three of the tested inhibitors, namely NSC697923 (NSC), bardoxolone methyl (BARM) and omaveloxolone (OMA) demonstrated broad-spectrum antiviral activity against VEEV and EEEV. Dose dependency and time of addition studies suggest that BARM and OMA exhibit intracellular and post-entry viral inhibition. Cumulatively, our studies indicate that inhibitors of the UPS-associated signaling pathways exert broad-spectrum antiviral outcomes in the context of VEEV and EEEV infection, supporting their translational application as therapeutic candidates to treat alphavirus infections.

## 1. Introduction

New World encephalitic alphaviruses, including Venezuelan Equine Encephalitis Virus (VEEV) and the closely related Eastern Equine Encephalitis Virus (EEEV), are classified as re-emerging viruses and category B select agents by the National Institutes of Health (NIH) and the Centers for Disease Control and Prevention (CDC). VEEV and EEEV belong to the family of Togaviridae and are positive-strand RNA viruses [1,2,3]. Equine Encephalitis disease occurs naturally in humans in many parts of the world annually due to transmission by infected mosquitoes. Infections have been recorded for several decades in the Americas, primarily associated with natural transmission by infected mosquito vectors [4,5,6,7,8,9]. VEEV and EEEV are also highly stable and retain infectivity as aerosols, which greatly increases the possibility of encephalitic disease in infected individuals. VEEV or EEEV exposures due to deliberate aerosol dissemination pose encephalitis concerns because of their ability to establish a quick infection in the central nervous system (CNS) through the olfactory neuron, by-passing the blood–brain barrier (BBB) [10,11,12,13,14]. The establishment of a robust productive infection in the CNS triggers a strong inflammatory response that impacts the integrity of the BBB and contributes to encephalitic disease [15,16,17,18]. There are currently no FDA-approved small molecule therapeutic strategies to treat VEEV or EEEV exposures.

Host-based signaling mechanisms play critical roles in the establishment of a productive VEEV and EEEV infection in in vitro and in vivo models, thus opening up the possibility of host-based proteins as broad-spectrum targets for therapeutic intervention [19,20,21,22,23,24]. We previously demonstrated that the ubiquitin proteasome system (UPS) is important for VEEV infection and inhibiting the UPS by small molecules elicits broad-spectrum antiviral activity against alphaviruses [25]. The UPS is also a critical requirement for several other acutely infectious viruses that possess epidemic/pandemic potential such as chikungunya virus, dengue virus and influenza virus, thus identifying this pathway as a broadly relevant target for epidemic/pandemic preparedness [25,26,27,28,29,30,31,32,33,34]. In addition to the UPS machinery itself, many signaling pathways that involve ubiquitination of specific signaling molecules have also been demonstrated to be viable targets for intervention against acutely infectious viruses. Notable among such signaling pathways with essential ubiquitination steps are the NFκB signaling pathway and the Nrf2 pathway, which are known to be targets for host–virus interaction for many acute viruses [34,35,36,37,38,39,40,41,42,43]. We previously demonstrated that the NFκB signaling pathways plays an important role in the establishment of a productive infection for VEEV and EEEV [36]. The wealth of data that exist regarding the important roles of the UPS, the NFκB and Nrf2 signaling pathways led us to hypothesize that inhibitors that interfere with UPS-mediated signaling events will exert an inhibitory outcome in the context of alphaviruses.

We tested our hypothesis using eight small molecules that are well documented in the literature as inhibitors of NFκB and Nrf2 signaling events by interfering with ubiquitination modification. Our data demonstrate that two inhibitors, omaveloxolone (OMA), and bardoxolone methyl (BARM) exhibit potent, broad-spectrum inhibitory potential against VEEV and EEEV in a cell-type-independent manner. Treatment of VEEV-TC83-infected cells with OMA also resulted in the inhibition of several proinflammatory cytokines, thus adding support to the utility of these small molecules as broad-spectrum inhibitors of New World alphaviruses.

## 2. Materials and Methods

### 2.1. Cell Culture

Vero African Green Monkey kidney epithelial cells (ATCC, CCL-81) were grown in Dulbecco’s Modified Eagle’s Medium (DMEM, Quality Biological, 112-013, 101CS, Gaithersburg, MD, USA) supplemented with 5% heat-inactivated fetal bovine serum (FBS), 1% penicillin and streptomycin (P/S) (Corning 30-003-CI, Corning, NY, USA), and 1% L-glutamine (Corning, 25-005-CI, Corning NY, USA). Human microglial cells, HMC3 (CRL-3304, ATCC, Manassas, VA, USA) and astroglial cells SVG-p12, (ATCC, CRL-8621) were cultured in Eagle’s Minimum Essential Medium with 10% FBS and 1% P/S. Cells were plated per well at a density of 1.5 × 10^5^ for 12-well plates, 5.0 × 10^4^ for 24-well plates. For 96-well plates, HMC3 and SVG-p12 cells were plated at 100 µL at 1.0 × 10^4^ or 200 µL at 2.0 × 10^4^ per well, and Vero cells were plated at 100 µL 5.0 × 10^4^ cells per well. All cell lines were maintained at 37 °C and 5% CO_2_ culture conditions.

### 2.2. Viruses and Viral Infection

Venezuelan Equine Encephalitis Virus (VEEV) TC83 strain was generated using a genomic clone that was kindly provided by Dr. Frolov (University of Alabama at Birmingham). VEEV Trinidad Donkey (TrD) strain and EEEV FL93 strains were kindly provided by Dr. Kehn-Hall (Virginia Polytechnic Institute and State University). All research activities involving select agents that are included in the manuscript were conducted at George Mason University’s Biomedical Research Laboratory with registration and compliance in accordance with Federal Select Agent regulations.

### 2.3. Inhibitors

The inhibitors that were utilized in this study were purchased from MedChemExpress (Monmouth Junction, NJ, USA); BARM (bardoxolone methyl/RTA 402, Cat. HY-13324), OMA (omaveloxolone/RTA 408, Cat. HY-12212), NSC (NSC697923, Cat. HY-13811), BAR (bardoxolone, Cat. HY-14909), P00 (P005091, Cat. 87 HY-15667), YH1 (YH239-EE, Cat. HY-12287), JHS (JHS-23, Cat. HY-13982), ML (ML-323, Cat. 88 HY-17543).

### 2.4. Drug Treatment and Plaque Assay

The treatment strategies for the small molecule inhibitors and the plaque assay methodology for quantification of virus infectious titer for VEEV and EEEV were carried out following procedures that are well described in the literature [19,20,21,22,23,24]. Briefly, cells were seeded at 1.0 × 10^4^ in 96-well plate or 5.0 × 10^4^ cells per well in 24-well plate. Following 24 h of culture at 37 °C and 5% CO_2_, cells were pre-treated for 1 h with inhibitors or the DMSO control (0.1%). For purpose of consistency, a DMSO control was included for all infections/treatment experiments at 0.1% unless stated otherwise [44,45,46]. Concentrations for each inhibitor vary in each experiment unless stated otherwise. Cultured cells were infected at a multiplicity of infection (MOI) of 0.1, unless stated otherwise, for 1 h with VEEV-TC83, VEEV-TrD or EEEV FL93. Cells were washed with PBS after infection and the inhibitor-containing media with the same concentrations as in pre-treatment were added back to the cells after 1 h of infection. Culture supernatants were collected at different time points (6 or 18 h post-infection) and analyzed by plaque assays.

For plaque assays, Vero cells were plated in 12-well plates at 1.5 × 10^5^ cells per well. Supernatant samples were diluted in DMEM from 10^1^ to 10^8^ and infection was carried out for each dilution as described above. At 1 h post-infection, 1 mL of a 1:1 solution of 1% agarose in distilled H_2_O with 2x Eagle’s Minimal Essential Medium was added to each well. Plates were allowed to solidify at room temperature and subsequently transferred to 37 °C, 5% CO_2_ culture condition for 48 h. At 48 h post-infection, plates were fixed with 10% formaldehyde overnight at room temperature. Approximately 24 h after fixation, the agar plugs were discarded and fixed cells were stained with 1% crystal violet in 20% methanol solution for 15 min. The plaques were counted for each plate and plaque forming units/mL (PFU/mL) for each sample was determined. The mean and standard deviation were determined using the average of 3 replicates for each sample.

### 2.5. Cell Viability Assay

Cell viability assay was performed on inhibitor-treated cells to quantify cytotoxicity using CellTiterGlo Cell Luminescent Viability Assay according to manufacturer’s instructions (Promega, G7570, Madison, WI, USA). As readout, the ATP level in cells was detected via luminescence, and percent viability was quantified relative to the DMSO control.

### 2.6. RNA Extraction and qRT-PCR Assay

The analysis of viral RNA by qRT-PCR was performed following methodologies that are published for VEEV and EEEV [19,20,21,22,23,24]. Briefly, cells were lysed with TriZol LS (ThermoFisher, Waltham, MA, USA), and total RNA was isolated from cells with the Direct-zol RNA miniprep kit (Zymo Research, Irvine, CA, USA) according to the manufacturer’s protocol. The intracellular viral RNA quantification was performed using the RNA UltraSenseTM One-step Quantitative RT-PCR System (Applied Biosystems, Waltham, MA, USA). The experiments were performed according to a standardized protocol using 20 µL of master mix using Verso 1-step RT-qPCR Mix with ROX (Fischer Science, Hamptom, NH, USA) and 5 µL of sample RNA, using VEEV-TC83 positive-strand Probe (5′-TGTTGGAAGGAAGATAAACGGCTACGC-3′), forward primer (5′-TCTGACAAGACGTTCCCAATCA-3′) and reverse primer (5′-GAATAACTTCCCTCCGACCACA-3′). The samples were heated at 50 °C for 20 min, 95 °C for 15 min, followed by 40 cycles of 95 °C (15 s) and 60 °C (60 s). The standard curve was determined using serial dilutions of isolated VEEV-TC83 RNA. RNA genomic copies were determined relative to a standard curve containing known amount of viral RNA. Intracellular RNA data were determined per 10,000 cells, while the extracellular RNA data were determined based on supernatant volume.

### 2.7. Negative Strand RT-qPCR

HMC3 cells were plated in a 12-well plate at a density of 1.5 × 10^5^ cells per well and maintained at a 37 °C and 5% CO_2_ culture conditions overnight. Cells were pre-treated with inhibitors OMA, BARM or DMSO control for 1 h, followed by infection with VEEV-TC83 at an Moi 0.1 for 1 h. Inhibitor-conditioning medium was added back after viral overlay was removed and cells were kept at 37 °C and 5% CO_2_ culture conditions. Six hours post-infection, supernatants and intracellular RNA were collected and stored at −80 °C. Viral intracellular RNA was extracted as previously described. cDNA was generated using a specific primer to negative-strand RNA for VEEV TC-83, which contained a T7 promoter sequence attached at the 5′ end (T7-TC83-Neg 5′-GCGTAATACGACTCACTATATCCGTCAGCTCTCTCGCAGG-3′). A high-capacity cDNA reverse transcription kit (4368814, ThermoFisher, Waltham, MA, USA) was used to generated the negative-strand cDNA per the manufacturer’s instructions. For qPCR of negative-strand viral RNA, forward primer specific to the T7 promoter sequence (5′-GCGTAATACGACTCACTATA-3′) and reverse primer specific to VEEV TC-83 (5′-CAGGTACTAGGTTTATGCGC-3′) were utilized. qPCR for detection of viral negative strand used thermal cycling conditions adapted from PowerUp SYBR Green (A25742, ThermoFisher Scientific, Waltham, MA, USA) per the manufacturer’s instructions: 1 cycle at 50 °C for 2 min, 1 cycle at 95 °C for 2 min, 40 cycles at 95 °C for 15 s, 60 °C for 15 s and 72 °C for 1 min using StepOnePlus™ Real Time PCR system (ThermoFisher Scientific, Waltham, MA, USA). The ΔΔCt method was used to determine the fold change compared to the DSMO average.

### 2.8. Luciferase and Bradford Protein Assay

HMC3 or SVGp12 cells were plated in 96-well plate at a density of 1.0 × 10^4^ cells per well and pre-treated for 1 h with selected inhibitors and DMSO control, then infected for one hour with VEEV-TC83, and then post-treated with the same inhibitors, DMSO control or cell culture media. At 18 hpi, supernatants were collected and cellular lysates were obtained using 1X Passive Lysis Buffer (E1941, Promega, Madison, WI, USA), and Nano-Glo Luciferase Assay System (N1130, Promega, Madison, WI, USA) was used to measure the luciferase activity per the manufacturer’s instructions. Aliquots of the lysates were mixed with Bradford Reagent (5000006, Bio-Rad, Hercules, CA, USA) per manufacturer’s instructions. A standard curve for total protein was established using bovine serum albumin (BSA, BP1600, FisherSci, Hamptom, NH, USA) diluted in Passive Lysis Buffer at concentrations of 1, 2.5, 5, 10, 20 μg/μL. Mock-infected cells were used to establish the limit of detection for the luciferase assays. Luminescence and absorbance were measured using a GloMax Promega plate reader (Promega, Madison, WI, USA). Intracellular luciferase was normalized to total μg of protein.

### 2.9. Proinflammatory Cytokine Quantification Assay

HMC3 cells were plated in 24-well plate at a density of 5.0 × 10^4^ cells per well and pre-treated for 1 h with selected inhibitors, DMSO control or cell culture medium. After 1 h, inhibitors were removed and cells were infected for 1 h with VEEV-TC83. Medium by itself, or with inhibitors or DMSO was added back to the cells after infection. Supernatants were collected at 6 and 18 h post-infection and stored at −80. The collected supernatants from the two timepoints were assayed with MSD V-PLEX Proinflammatory Panel Human Kit (Cat. K15049D-2) as duplicates for 10 cytokines: IFN-γ, IL-1β, IL-2, IL-4, IL-6, IL-8, IL-10, IL-12p70, IL-13 and TNF-α. The assay was performed following the manufacturer’s protocol and read using MESO QuickPlex SQ 120 (MesoScale Discovery, Gaithersburg, MD, USA).

### 2.10. Statistics

All quantifications were performed by incorporating data obtained from triplicate samples unless indicated otherwise. Error bars in all figures indicate standard deviations. Plaque assay, qRT-PCR and ELISA data calculations were performed using Microsoft Excel. Graphs and p-values were designed and calculated on GraphPad Prism version 9.2.0 for Windows 10 or 9.4.0 for MacOS. Significance values are indicated using One-way ANOVA with Dunnett’s post-test using asterisks as * *p* < 0.05, ** *p* < 0.01, *** *p* < 0.001, **** *p* < 0.0001, or using unpaired two-tailed *t*-test * *p* < 0.05, ** *p* < 0.01, *** *p* < 0.001, **** *p* < 0.0001.

## 3. Results

### 3.1. Inhibitors of UPS-Mediated Signaling Events Decrease VEEV-TC83 Load in Vero Cells

**Figure 1:**
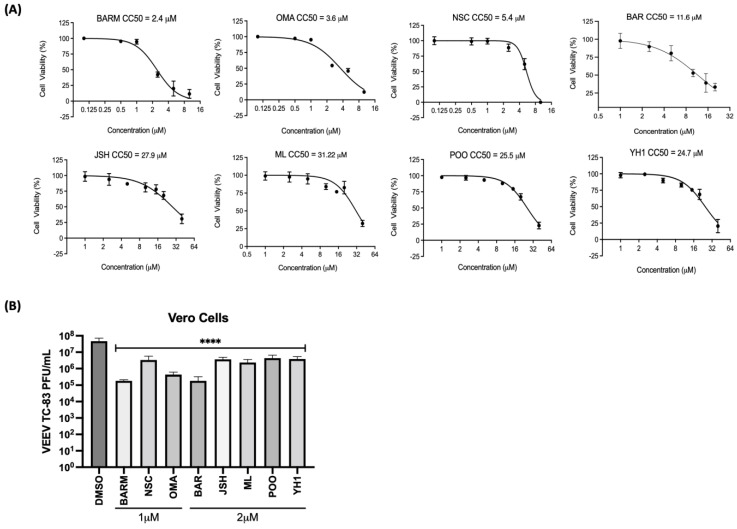
Inhibitors of UPS-mediated signaling events decrease Venezuelan Equine Encephalitis Virus (VEEV) TC83 infectious titer in Vero cells. (**A**) Vero cells were pre-treated with the inhibitor as indicated at increasing concentrations, toxicity was quantified at 24 h post-treatment using Cell Titer Glo and luciferase values reported as percent viability relative to the DMSO control. (**B**) Cells were treated with either 1 or 2 µM concentrations of each inhibitor for 1 h, after which they were infected with VEEV-TC83 (MOI: 0.1). Post-infection, the cells were treated with the inhibitor-containing media or media with DMSO and incubated at 37 °C, 5% CO_2_ for 18 h, after which infectious titer in the supernatants was quantified by plaque assay. Statistical analysis was performed using One-way ANOVA with Dunnett’s post-test. **** *p* < 0.0001.

Eight candidate inhibitors that target UPS-mediated signaling events, with an emphasis on NFkB signaling and Nrf2 signaling, were chosen to analyze their potential inhibitory effects on alphavirus multiplication using Venezuelan Equine Encephalitis Virus (VEEV) TC-83, the test pathogen (Table 1). As the first step, the cytotoxicity of these compounds was assessed in Vero cells and 50% cytotoxic concentration (CC^50^) values were determined for each inhibitor, and DMSO (0.1%) was included as the vehicle control (Figure 1A). The cells were incubated for 24 h with media containing increasing concentrations of each inhibitor and cell viability quantified by CellTiterGlo assay. The CC^50^ values for BARM (2.4 µM), OMA (3.5 µM), NSC (5.4 µM), BAR (11.6 µM), YH1 (24.7 µM), P00 (25.5 µM), JSH (27.9 µM) and ML (31.2 µM) were determined. To assess the inhibitory potential of each inhibitor, Vero cells were pre-treated with the inhibitor at 1 µM (BARM, OMA, NSC) or 2 µM (BAR, JSH, P00, YH1, ML) for 1 h, after which the cells were infected with VEEV-TC83 (multiplicity of infection (MOI): 0.1) for 1 h at 37 °C. After 1 h to permit infection, the virus overlay was replaced with the inhibitor-containing media and cells were maintained at 37 °C for 18 h. The culture supernatants were collected at 18 h post-infection and viral load quantified by plaque assay (Figure 1B). The data demonstrate that all the chosen inhibitors exerted an inhibitory effect, albeit to varying degrees, with BARM, BAR and OMA demonstrating a >2 log decrease as compared to the DMSO control. NSC, P00, ML, JHS and YH1 demonstrated a >1 log reduction in the TC-83 titer as compared to the DMSO control. Collectively, our initial assessment of the chosen inhibitors in Vero cells add support to the significance of UPS-mediated signaling events for the establishment of a productive VEEV infection.

### 3.2. UPS-Mediated Signaling Inhibitors Demonstrate Inhibition of VEEV-TC83 in Human Astroglial (SVG-p12) and Microglial (HMC3) Cells

**Figure 2:**
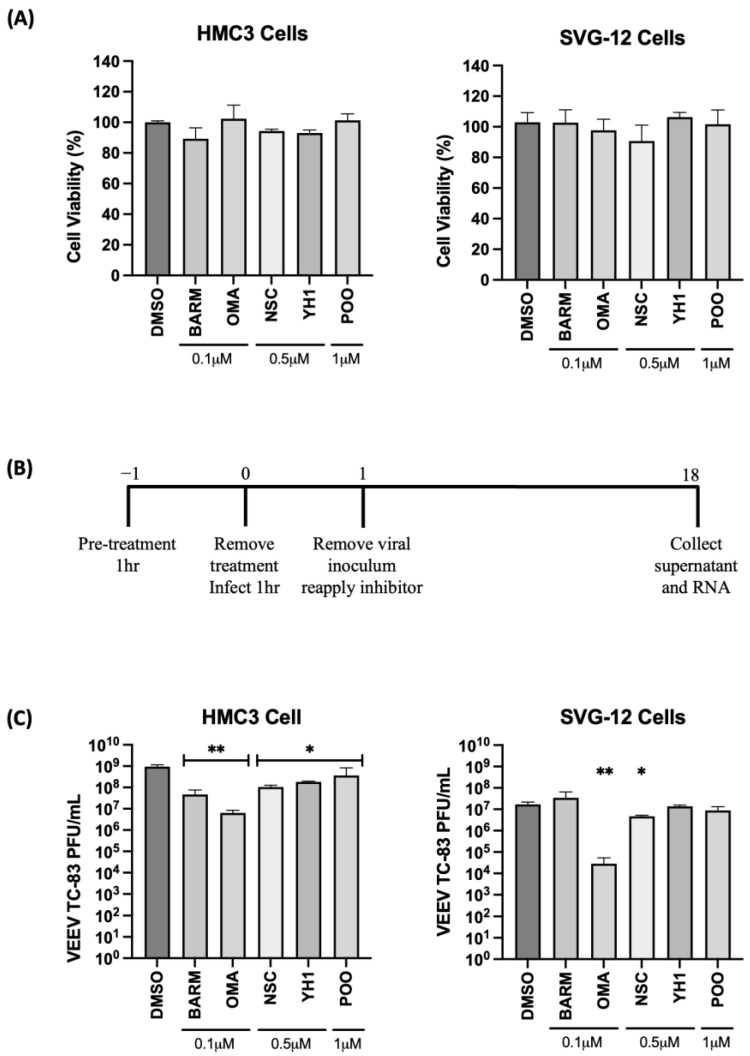
Inhibitors of UPS-mediated signaling inhibit VEEV-TC83 in infected microglial (HMC3) and astroglial (SVG-p12) cells. (**A**) HMC3 and SVG-p12 cells were plated in 96-well format plates and treated with inhibitors at the indicated concentrations. DMSO-treated cells were maintained as vehicle-alone controls. Treated cells were maintained at 37 °C, 5% CO_2_ for 24 h, and cell viability was quantified by Cell Titer Glo assay. (**B**) Schematic of infection. (**C**) HMC3 and SVG-p12 cells were pre-treated for 1 h with inhibitors or DMSO, followed by an infection with VEEV-TC83 for 1 h. Inhibitor-containing medium was added back to the cells after removal of the viral overlay and cells were incubated at 37 °C, 5% CO_2_. Eighteen hours post-infection, supernatants were collected and assessed by plaque assay for viral load. Infectious virus titers are reported as plaque-forming units (PFU)/mL. Statistical analysis was performed using One-way ANOVA with Dunnett’s post-test. * *p* < 0.05, ** *p* < 0.01.

VEEV and EEEV are known to infect cells of the CNS that contribute to the encephalitic phenotype in infected individuals. Thus, we determined if the inhibitors that demonstrated successful antiviral outcomes in Vero cells were also able to elicit robust inhibition of VEEV in human-derived cells of the CNS, specifically, astroglial (SVG-p12) and microglial (HMC3) cells. OMA, BARM, NSC, YH1 and P00 were selected for further testing in HMC3 and SVG-p12 cells. The cells were independently treated with OMA (0.1 µM), BARM (0.1 µM), NSC (0.5 µM), YH1 (0.5 µM), P00 (1 µM) and DMSO (0.1%) for 24 h and cytotoxicity assessed by CellTiterGlo assay (Figure 2A). The data demonstrate that >90% cell viability can be observed in all cases at selected concentrations. CC^50^ data for all five inhibitors in HMC3 and SVG-p12 cell types are included as supplemental data (Figure S1). The antiviral potential of these inhibitors in the two cell types was next assessed by preincubating the cells with inhibitor-containing media for 1 h, infection by VEEV-TC83 for 1 h and post-treatment of infected cells with inhibitors. DMSO was maintained as the vehicle-alone control. At 18 h post-infection, cell culture supernatants were collected and viral titers were quantified by plaque assay (Figure 2B,C).

**Figure 3:**
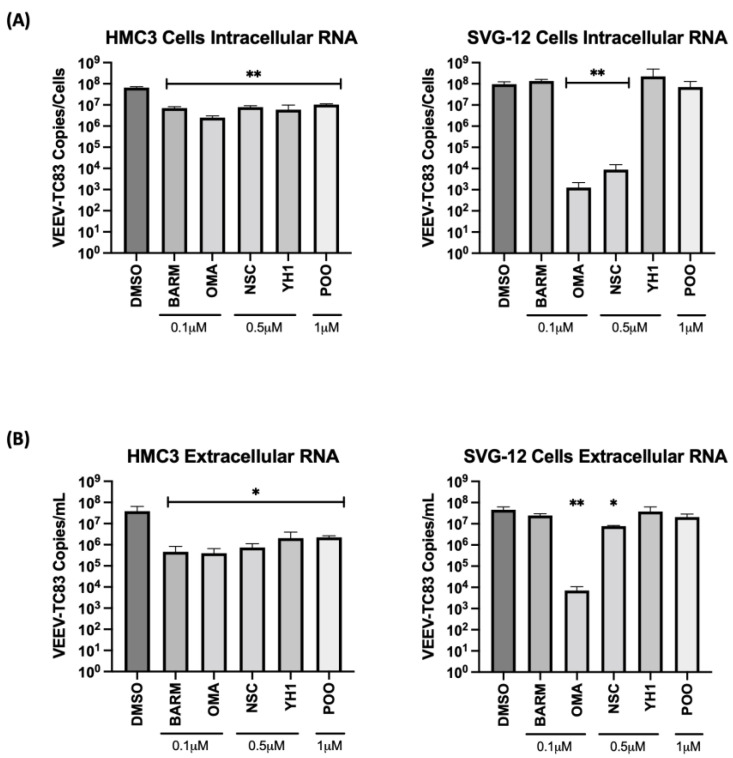
UPS-mediated signaling inhibitors decrease viral RNA in the VEEV-TC83-infected culture supernatants and in infected HMC3 and SVG-p12 cells. HMC3 cells and SVG-p12 cells were seeded in a 96-well plate at a density of 10,000 cells per well. Cells were pre-treated for 1 h with inhibitors or DMSO, followed by an infection with VEEV-TC83 for 1 h. Inhibitor-containing media or the corresponding DMSO control media were added back to cells post-infection and cells were incubated at 37 °C, 5% CO_2_. Eighteen hours post-infection, supernatants were collected and RNA was isolated for qRT-PCR analysis. Cells were lysed and intracellular RNA was isolated. The viral genomic copy number for VEEV-TC83 in the extracted RNA was quantified by qRT-PCR for (**A**) intracellular RNA and (**B**) extracellular (supernatant) viral RNA quantification. Statistical analysis was performed using One-way ANOVA with Dunnett’s post-test. * *p* < 0.05, ** *p* < 0.01.

A fraction of the supernatant was used for RNA isolation to quantify extracellular viral RNA levels. The cells were then lysed and total RNA obtained to quantify the impact of the inhibitors on intracellular viral RNA levels (Figure 3A,B). In HMC3 cells, all five of the selected inhibitors decreased infectious virus titer when compared to the vehicle-alone control, while in SVG-p12 cells, only OMA and NSC demonstrated statistically significant inhibition (Figure 2C).

The viral RNA levels in the cell culture supernatants and infected cells were quantified by qRT-PCR to ascertain the effect of the inhibitors on the extracellular and intracellular viral RNA levels, respectively. In the context of HMC3 cells, OMA treatment reduced the intracellular viral RNA level by >1 log, while NSC, BARM, YH1 and P00 treatment reduced the intracellular viral RNA by approximately 1 log (Figure 3A). In the context of SVG-p12 cells, OMA demonstrated a >4 log decrease and NSC showed a >3 log decrease in intracellular viral RNA, while BARM, YH1 and P00 did not demonstrate a statistically significant decrease. Quantification of extracellular viral RNA in HMC3 cells showed a uniform decrease of >1 log for all inhibitors (Figure 3B), agreeing with the trend seen with intracellular viral RNA. In the SVG-p12 cells, while OMA consistently reduced extracellular viral RNA by >3 logs (Figure 3B), trending similar to the intracellular viral RNA (Figure 3A), the extracellular viral RNA reduction by NSC was less significant as compared to the intracellular RNA. Cumulatively, the studies in CNS-relevant cell types, namely the astroglial and microglial cells, demonstrate that OMA demonstrated consistent inhibition of VEEV-TC83 in a cell-type-independent manner with reduction observed at the level of infectious titer and viral RNA. BARM and NSC continue to show promise as inhibitors of VEEV-TC83 in these cell lines, albeit at less robust levels than OMA. Additionally, CC^50^ and effective concentration at 50% percent (EC^50^) further confirmed OMA, BARM and NSC as being effective inhibitors (Figure S2).

### 3.3. UPS Signaling Inhibitors Decrease VEEV-TC83 in a Dose-Dependent Manner

**Figure 4:**
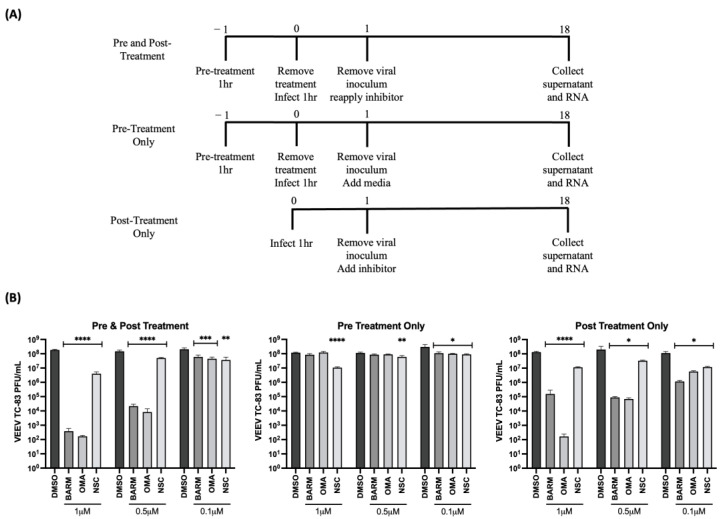
OMA, BARM and NSC demonstrated dose-dependent decrease in infectious titers of VEEV-TC83 when inhibitors were provided pre- and post-infection. (**A**) Schematic of infection and inhibitor treatment strategies; (**B**) HMC3 cells were treated with the treatment methods as indicated at increasing concentrations of OMA, BARM and NSC. Infection was performed with VEEV-TC83 for 1 h, after which the media with or without inhibitors or DMSO was added back to cells. Supernatants were obtained at 18 h post-infection and infectious titer quantified by plaque assay. Statistical analysis was performed using One-way ANOVA with Dunnett’s post-test. * *p* < 0.05, ** *p* < 0.01, *** *p* < 0.001, **** *p* < 0.0001.

OMA, BARM and NSC were selected to further assess dose dependency of their inhibitory outcomes using HMC3 cells as the cell type of choice. Viral inhibition was queried under three different treatment conditions as shown in the schematic (Figure 4A), namely pre-treatment only, pre- and post-treatment and post-treatment only. Under each condition, the inhibitors were tested at increasing concentrations (0.1 µM, 0.5 µM and 1.0 µM) while still staying within the cytotoxic concentration that resulted in >90% survival (Figure S1). Although all three inhibitors showed a dose-dependent reduction in viral infectious titer in pre- and post-treatment, and in post-treatment-only strategies (Figure 4B), OMA and BARM showed a significant reduction in infectious titer compared to NSC. At 0.1 µM concentration, the three inhibitors showed a ~1 log reduction in the pre- and post-treatment condition. OMA and BARM showed a >5 log reduction at the 1 µM concentration in the pre- and post-treatment condition. When the same groups are compared at the highest concentration tested (1.0 µM), OMA consistently exerted strong inhibition, while BARM showed less inhibition in the post-treatment condition. The pre-treatment-only condition did not result in a robust reduction in viral load (<1 log) although statistical significance could be observed. Interestingly, when the post-treatment-only condition at the lowest concentration (0.1 µM) for the three compounds was compared to the pre- and post-treatment condition, the former resulted in more inhibition but with lower statistical significance. Overall, OMA and BARM demonstrated clear dose dependency in HMC3 cells, with the pre- and post-treatment strategy eliciting strong, statistically significant inhibition of infectious titers at MOIs of 0.1 and 1 (Figure S3).

**Figure 5:**
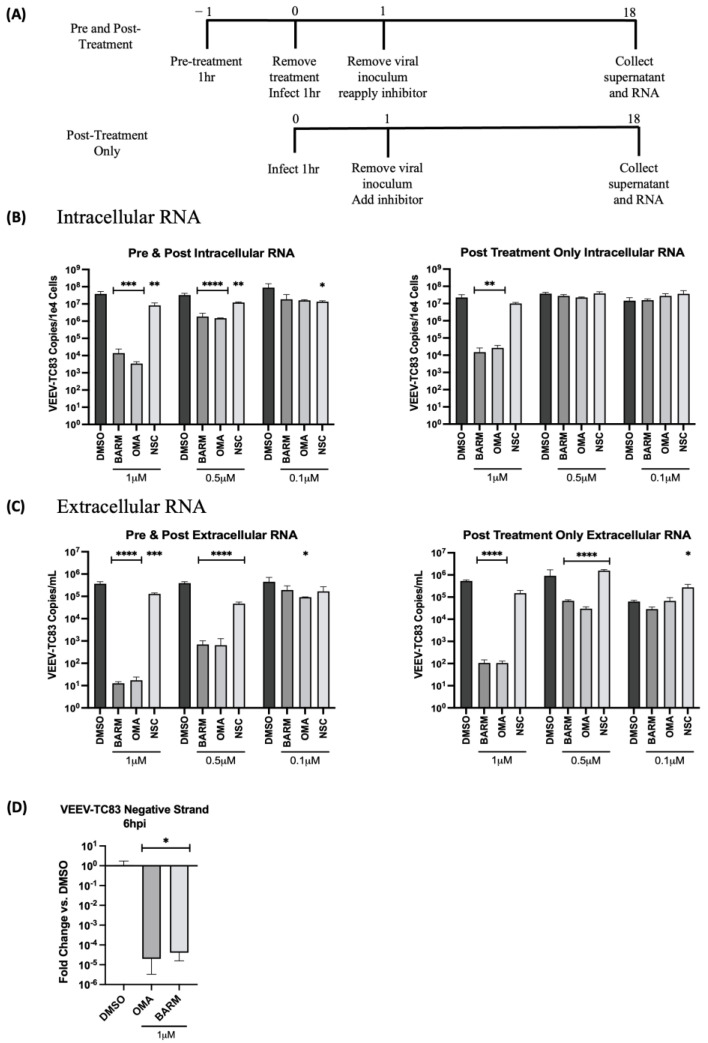
OMA, BARM and NSC showed a dose-dependent decrease in VEEV-TC83 RNA when inhibitor was present pre- and post-infection. (**A**) HMC3 cells were treated with the inhibitor either pre- and post-infection or post-infection only at different concentrations as indicated. Following infection for 1 h, the viral overlay was replaced with media containing the inhibitors. Viral RNA from culture supernatants and cells were collected at 18 h post-infection. (**B**) Intracellular viral RNA load and (**C**) extracellular viral RNA levels were analyzed by qRT-PCR and data reported as genomic copies per cell (intracellular) or per mL (extracellular). (**D**) Quantitative analysis of negative-strand RNA was performed with OMA and BARM. Statistical analysis determined using One-way ANOVA with Dunnett’s post-test. * *p* < 0.05, ** *p* < 0.01, *** *p* < 0.001, **** *p* < 0.0001.

The dose dependency of these inhibitors was also assessed at the level of intracellular and extracellular viral RNA by qRT-PCR in HMC3 cells. This analysis was restricted to only the pre- and post-treatment and the post-treatment-alone strategies (Figure 5A). The concentrations of OMA, BARM and NSC were maintained the same as described above for infectious viral titers. The analysis of intracellular RNA revealed that at the highest concentration tested (1 µM), the decrease in viral RNA in OMA, BARM and NSC treatments were highly comparable, producing a >3 log drop for OMA and BARM and a ~1 log drop for NSC (Figure 5B). At the lowest concentration tested (0.1 µM), there was no robust inhibitory effect noted for any of the inhibitors. At the mid-level concentration (0.5 µM), OMA and BARM elicited a >2 log inhibition, which was statistically significant. The overall trend for these three inhibitors, at these three concentrations, was fairly comparable when the extracellular viral RNA was quantified (Figure 5C). The lowest concentrations of the three compounds did not show robust inhibition, while very strong inhibition (>4 log) was seen for OMA and BARM in both conditions. Overall, the assessment of the dose dependency of the three inhibitors at the infectious titer (Figure 4) and viral RNA levels (Figure 5) indicate that pre- and post-treatment with OMA and BARM produce a clear, dose-dependent decrease in VEEV-TC83 infectious titer and viral RNA in HMC3 cells. Additionally, OMA and BARM at 1 µM concentration show a statistically significant reduction in viral negative-strand RNA levels in VEEV-TC83-infected HMC3 cells at 6 h post-infection (Figure 5D).

### 3.4. OMA, BARM and NSC Exhibit Differential Inhibitory Impact on Proinflammatory Cytokines in VEEV-TC83-Infected HMC3 Cells

**Figure 6:**
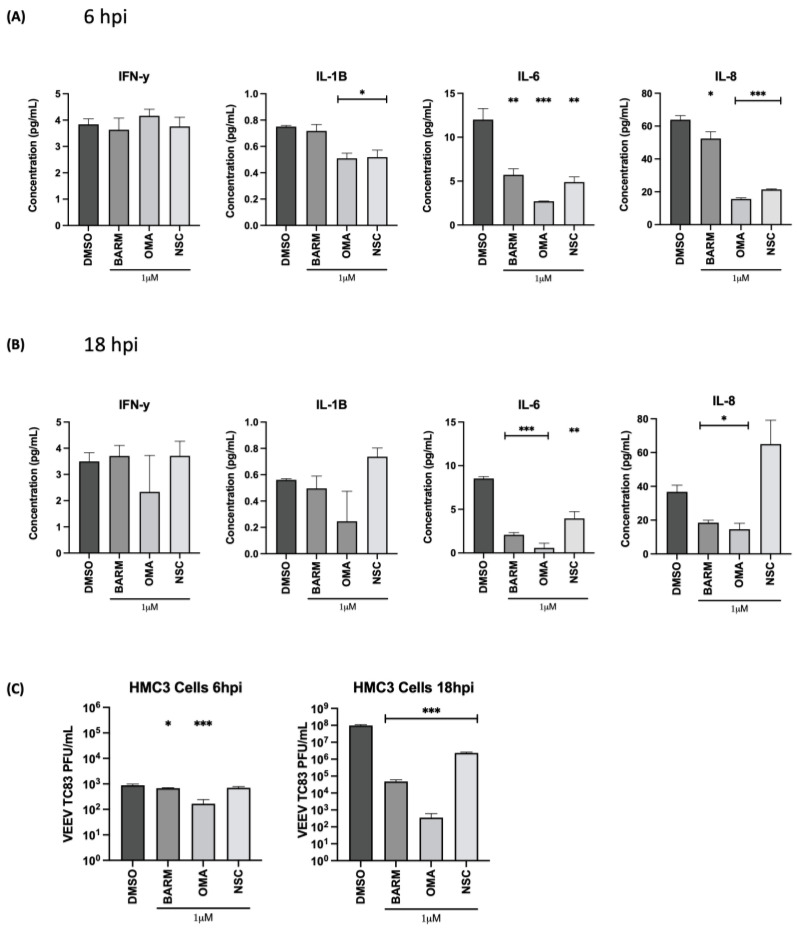
Effect of OMA, BARM and NSC treatment on expression of proinflammatory cytokines in the supernatants of VEEV-TC83-infected HMC3 cells. HMC3 cells were seeded in a 96-well plate format at a cell density of 10,000 cells per well. Cells were pre-treated for 1 h with inhibitors or DMSO, followed by an infection with VEEV-TC83. Following the infection, cells were treated again with the inhibitors or with DMSO. Culture supernatants were collected at 6 and 18 h post-infection (**A**,**B**), which were used for assessment of proinflammatory cytokine levels using MSD V-PLEX Proinflammatory Human Kit. Using this kit, 10 cytokines were quantified, namely IFN-γ, IL-1β, IL-2, IL-4, IL-6, IL-8, IL-10, IL-12p70, IL-13 and TNF-α. The results were reported as pg/mL for each cytokine at each timepoint. (**C**) The same culture supernatants that were used for the proinflammatory cytokine quantification were also used to quantify infectious viral titer by plaque assay to establish infectivity correlates for each time point and each inhibitor. Statistical analysis was carried out using *t*-test * *p* < 0.05, ** *p* < 0.01, *** *p* < 0.001.

OMA, BARM and NSC are known to modulate the NFκB signaling cascade, which is an important modulator of proinflammatory cytokine expression. Therefore, the impact of OMA, BARM and NSC treatment on proinflammatory cytokine levels in the context of the pre- and post-infection treatment of infected HMC3 cells was analyzed by multiplexed ELISA. The supernatants from cells treated with the inhibitors or with the vehicle-alone control were obtained at 6 h and 18 h post-infection and quantified for the levels of 10 inflammatory cytokines (IFN-γ, IL-1β, IL-2, IL-4, IL-6, IL-8, IL-10, IL-12p70, IL-13 and TNF-α) using the V-PLEX proinflammatory human cytokine array (Mesoscale). At 6 h post-infection, OMA- and NSC-treated cells had an inhibitory effect on IL-1β, IL-6 and IL-8, while BARM inhibited IL-6 and IL-8 levels (Figure 6A). There was no significant change in IFNγ levels with any of the inhibitors. Similarly, at 18 h post-infection, OMA had an inhibitory effect on IL-1β, IL -6 and IL-8. BARM showed a more inhibitory effect compared to the control at 18 h post-infection with IL-6 and IL-8 but not for IL-1β (Figure 6B). At 18 h post-infection, NSC exerted inhibitory activity only on IL-6 (Figure 6B). The 6 and 18 h supernatant samples were also evaluated by plaque assay to measure the corresponding viral load at those time points (Figure 6C) and, as expected, the viral load was lower at the 18 h time point in the inhibitor-treated samples. The inhibitory activities of the three compounds on the other proinflammatory cytokines are included in the supplemental data (Figure S4). Overall, at the early and the later time points tested, OMA exerted an inhibitory effect on three major proinflammatory cytokines, IL-1β, IL-6, IL-8, and BARM demonstrated an inhibitory effect against IL-6 and IL-8 in VEEV-TC83-infected HMC3 cells.

### 3.5. OMA, BARM and NSC Exert Broad-Spectrum Viral Inhibitory Activity against Virulent Strains of VEEV and EEEV in HMC3 Cells

**Figure 7:**
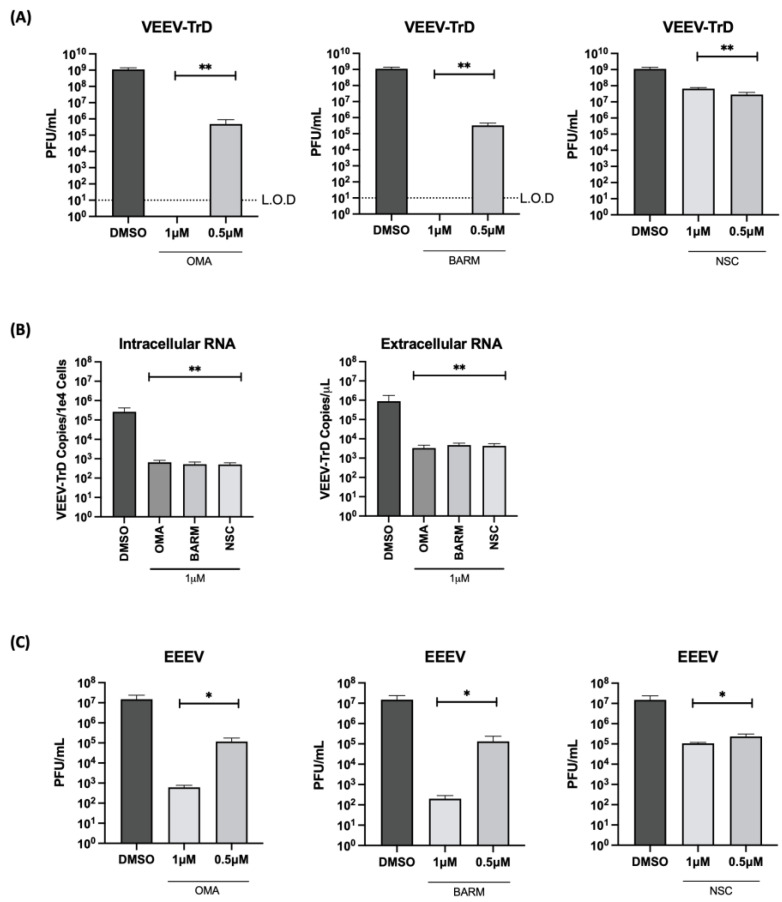
OMA, BARM and NSC demonstrate broad-spectrum inhibition of virulent strains of VEEV and EEEV. HMC3 cells were seeded in a 96-well plate and pre-treated for 1 h with inhibitors (0.5 µM) or DMSO, followed by an infection with VEEV-TrD or EEEV FL93 (MOI: 0.1). After infection, the cells were post-treated with the inhibitors or with DMSO-containing medium. (**A**,**C**) Culture supernatants were collected at 18 h post-infection and assessed by plaque assay. (**B**) RNA was isolated from VEEV-TrD-infected HMC3 cells for qRT-PCR analysis. Cells were lysed for intracellular RNA isolation and supernatant was collected for extracellular RNA. The viral genomic copy number for VEEV-TrD in the extracted RNA was quantified by qRT-PCR for intracellular RNA and extracellular (supernatant) viral RNA quantification, data reported as genomic copies per cell (intracellular) or per µL (extracellular). Infectious titer for each virus is reported as plaque-forming units (PFU) per mL. Limit of detection (L.O.D) is included for panel A. Statistical analysis determined using One-way ANOVA with Dunnett’s post-test. * *p* < 0.05, ** *p* < 0.01.

The inhibitory potential of the three inhibitors against virulent strains of VEEV (VEEV-TrD) and Eastern Equine Encephalitis Virus (EEEV-FL93) were analyzed in HMC3 cells (Figure 7). The cells were pre- and post-infection treated with the inhibitors at 0.5 µM concentration and the effect on infectious viral titer was quantified at 18 h post-infection. With VEEV-TrD, a >2 log inhibition was observed with all three inhibitors, while EEEV, BARM and OMA exhibited a much stronger inhibition (>3 logs) than NSC, which exerted an inhibition of ~2 logs. Viral load analysis of VEEV-TrD with the three inhibitors at 1 µM concentration showed a >3 log reduction of both extracellular and intracellular RNA. Dose dependency of these inhibitors was also noted in the context of VEEV and EEEV infections (Figure 7A,C). Cumulatively, the data support the potential of these inhibitors to exert a broad-spectrum antiviral activity against virulent strains of New World alphaviruses.

## 4. Discussion

VEEV and EEEV are New World alphaviruses that are transmitted by mosquito vectors and contribute to disease in humans in the Americas. These viruses are highly stable and infectious as aerosols, in which case the encephalitic outcomes are prominent in the infected individuals, leading to higher rates of mortality than the natural transmission by mosquitoes. FDA-approved therapeutic strategies are not available to treat VEEV or EEEV infection.

Host-based inhibitors offer important advantages over virus-targeted inhibitors by decreasing the potential for the development of resistance and having a higher probability to exert broad-spectrum inhibitory outcomes in the context of closely related viruses. In this effort, we focused on a small selection of inhibitors that are known to influence the UPS-mediated signaling events in human host cells. Ubiquitination is an important post-translational modification that is mediated by the ubiquitin proteasome system, which includes enzymatic activities of several ubiquitin transferases and deubiquitinases [58]. Targeted protein degradation by the proteasome is also an important method for cell regulation that is dependent on the activity of the proteasome and the ubiquitination/deubiquitination enzymes. This is a multi-step process that is primarily carried out by E1 activating enzymes, E2 conjugating enzymes and E3 ubiquitin ligases, which ultimately leads to the proteasomal degradation of the ubiquitinated target by the 26S proteasome [58,59,60,61]. UPS is responsible for the degradation of targeted substrates (e.g., misfolded/unfolded proteins) and also functions to regulate many fundamental cellular processes such as stress response, signal transduction and transcriptional activation [58,59,60,61,62]. Many viral proteins are also regulated by differential ubiquitination and deubiquitination, thus suggesting that the associated enzymatic machinery can be targeted therapeutically to achieve virus inhibition. This has been documented for several viral proteins such as the NS1, NS3 and NS4B proteins of dengue virus [63]. The NS3 protease of dengue virus has been shown to interact with the E3 ligase, TRIM 69. Targeting the E3 ligase Cullin 2 exerts an inhibitory effect on dengue virus, adding support to the potential of these enzymes to be targeted to achieve viral inhibitory outcomes [64]. The Ebola virus protein VP35 is known to be ubiquitinated, which is required for the regulation of viral transcription and assembly [65]. The ubiquitination of VP40 has been demonstrated to be important for filovirus budding and egress [65,66,67,68]. In the context of New World alphaviruses, it has been demonstrated that inhibition of the proteasome function using the FDA-approved small molecule, bortezomib, exerted robust viral inhibitory activity against VEEV, by potentially impacting the ubiquitination status of the capsid protein [25]. Viral proteins also differentially regulate the availability and functionality of the UPS enzymatic machinery as evidenced in the context of chikungunya virus where the nsP2 protease downregulates an E2 conjugating enzyme [69]. Needless to say, there is a growing body of evidence in the literature that the UPS and the associated enzymatic machineries play critical roles in the establishment of productive infections in the context of several acutely infectious, enveloped RNA viruses, thus identifying them as valuable targets for therapeutic intervention.

Several host signaling pathways also include modification of critical signaling proteins to achieve their intended cell response outcomes [70,71]. The NFκB signaling cascade is a central mediator of multiple host response events including cell growth, multiplication and apoptosis [70,71,72,73,74,75]. NFκB signaling requires the nuclear translocation of p65, which is restricted in the cytoplasm in non-stimulated conditions by IκBα [75]. Upon activation of the NFκB cascade, IκBα is phosphorylated by IKKβ kinase, and ubiquitinated on K48, which targets IκBα for degradation. This targeted degradation of IκBα is required for the nuclear translocation of p65, which acts to regulate the transcription of key response genes [75]. The NFκB cascade has been shown to play an important role in New World alphavirus infections because inhibition of the IKKβ kinase by small molecule inhibitors exerted a broad-spectrum inhibitory activity against VEEV and EEEV [36,73]. It has also been demonstrated that IKKβ kinase can phosphorylate the VEEV non-structural protein 3 (nsP3) and this phosphorylation event is also important for infection [76]. While the role of the phosphorylation by the NFκB signaling cascade has been shown, the impact of modulating the ubiquitination status of the NFκB signaling cascade on alphavirus infections has not been looked into. The data included in this manuscript demonstrate that modulation of the ubiquitination-dependent signaling aspect of the NFκB cascade can also exert a broad-spectrum inhibitory effect against alphaviruses. This step, however, is downstream of IKKβ activation and hence, from a mechanistic perspective, is likely to involve alternate host and/or viral targets than those demonstrated earlier such as the VEEV nsP3 protein. It is more likely that the ubiquitination/deubiquitination machinery that is likely targeted by the highly effective small molecules OMA, BARM and NSC are critical enablers for the establishment of productive alphavirus infection. However, the data included in this study also demonstrate inhibition of proinflammatory cytokines in the context of inhibitor treatment, which is likely to be directly related to the inhibition of the nuclear translocation of p65 and the lack of activation of gene expression of proinflammatory genes such as IL-1β, IL-6 and IL-8.

The Nrf2 signaling pathway is well documented as playing important innate immune roles in cells including protective responses against oxidative stress and inflammation. VEEV infection has been shown to result in an increase in reactive oxygen species leading to mitochondrial damage and oxidative stress [77,78]. The Nrf2 signaling pathway is a target for multiple viruses including dengue virus [79,80], SARS-CoV-2 [81], hepatitis viruses [82], influenza virus [38] and Ebola virus [39,40,41,42], thus suggesting that this pathway can be targeted by therapeutic candidates to achieve a decrease in viral and/or inflammatory loads. Small molecule inhibitors that target the Nrf2 signaling pathway such as coumarin have been shown to exert an inhibitory effect in the context of alphavirus infections, specifically chikungunya virus [83]. The data included in this manuscript support the idea that the activation of Nrf2 signaling by small molecules can have broad-spectrum inhibitory outcomes against alphaviruses and also elicit protective outcomes by decreasing proinflammatory cytokine levels.

Of high significance in the context of encephalitic alphaviruses is the damage to the blood–brain barrier (BBB), and inflammation has been heavily implicated in BBB disruption. For a therapeutic candidate to be effective in an encephalitic disease state, the ability to reduce viral and inflammatory load will be an important requirement. Modulating Nrf2 levels at the BBB in the context of proinflammatory states such as diabetes and intracerebral hemorrhage has been shown to have positive outcomes [84,85], thus presenting the attractive possibility of OMA and BARM functioning in the protection of BBB integrity and decreasing the inflammatory load across the BBB during alphavirus infections.

## 5. Conclusions

Cumulatively, the data presented in this manuscript add support to the importance of UPS-mediated signaling events in the establishment of a productive infection by New World alphaviruses in cells of the CNS. The modulation of ubiquitination events in the NFκB and Nrf2 signaling pathways by small molecule inhibitors exert broad-spectrum inhibition of New World alphaviruses. It remains an attractive possibility that alphavirus proteins may be ubiquitinated/deubiquitinated by the same enzymatic machinery that results in ubiquitination modifications of host target proteins in the NFκB and Nrf2 signaling cascades, and hence, these small molecules may also have conserved viral targets.

## Figures and Tables

**Table 1:** Inhibitors of UPS-mediated signaling pathways.

Name of Inhibitor	Abbreviation	Mechanisms and Targets	References
Bardoxolone methyl	BARM	NFkB inhibitor, Nrf2 activator	[41,42,43,47]
Bardoxolone	BAR	IKK inhibitor, Nrf2 activator	[41,42,43,47]
Omaveloxolone	OMA	NFkB inhibitor, Nrf2 activator	[48,49]
NSC697923	NSC	NFkB inhibitor	[50,51,52]
YH239-EE	YH1	NFkB inhibitor, Nrf2 activator	[53]
P005091	P00	Ubiquitin-specific protease 7 inhibitor	[54]
JSH-23	JSH	NFkB pathway p65 translocation inhibitor	[55,56]
ML-323	ML	UPS1-UAF1 deubiquitinase complex inhibitor	[49,57]

## Supplementary Materials

The following supporting information can be downloaded at: https://www.mdpi.com/article/10.3390/v15030655/s1, Figure S1: Cell Viability of Selected 5 Inhibitors in Human Microglilal (HMC3) and Astroglial (SVG-p12) cells; Figure S2: Inhibitors of UPS-mediated signaling pathway inhibit VEEV-TC83 in Infected Human Microglilal (HMC3) and Astroglial (SVG-p12) cells; Figure S3: OMA, BARM, and NSC demonstrate inhibition of VEEV-TC83 infectious titer at Moi 1; Figure S4: Effect of OMA, BARM, and NSC treatment on expression.
