# Supplementary material for: Inhibitors of the Ubiquitin-Mediated Signaling Pathway Exhibit Broad-Spectrum Antiviral Activities against New World Alphaviruses
Source: Viruses. 2023 Feb 28;15(3):655. doi: 10.3390/v15030655 (PMC10059822; doi:10.3390/v15030655)

Figure S1. Cell viability of Selected 5 inhibitors in Human microglial (HMC3) and astroglial (SVG-p12) cells.

(A)

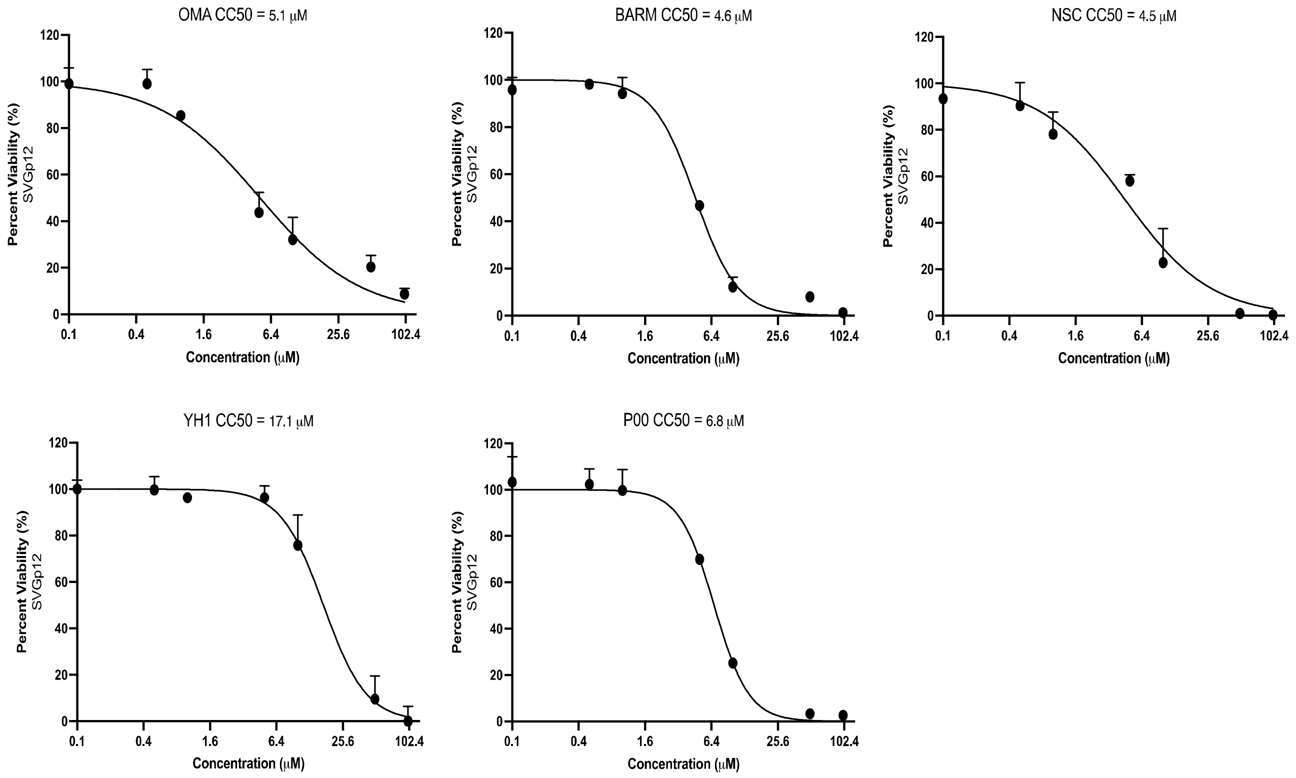

(B)

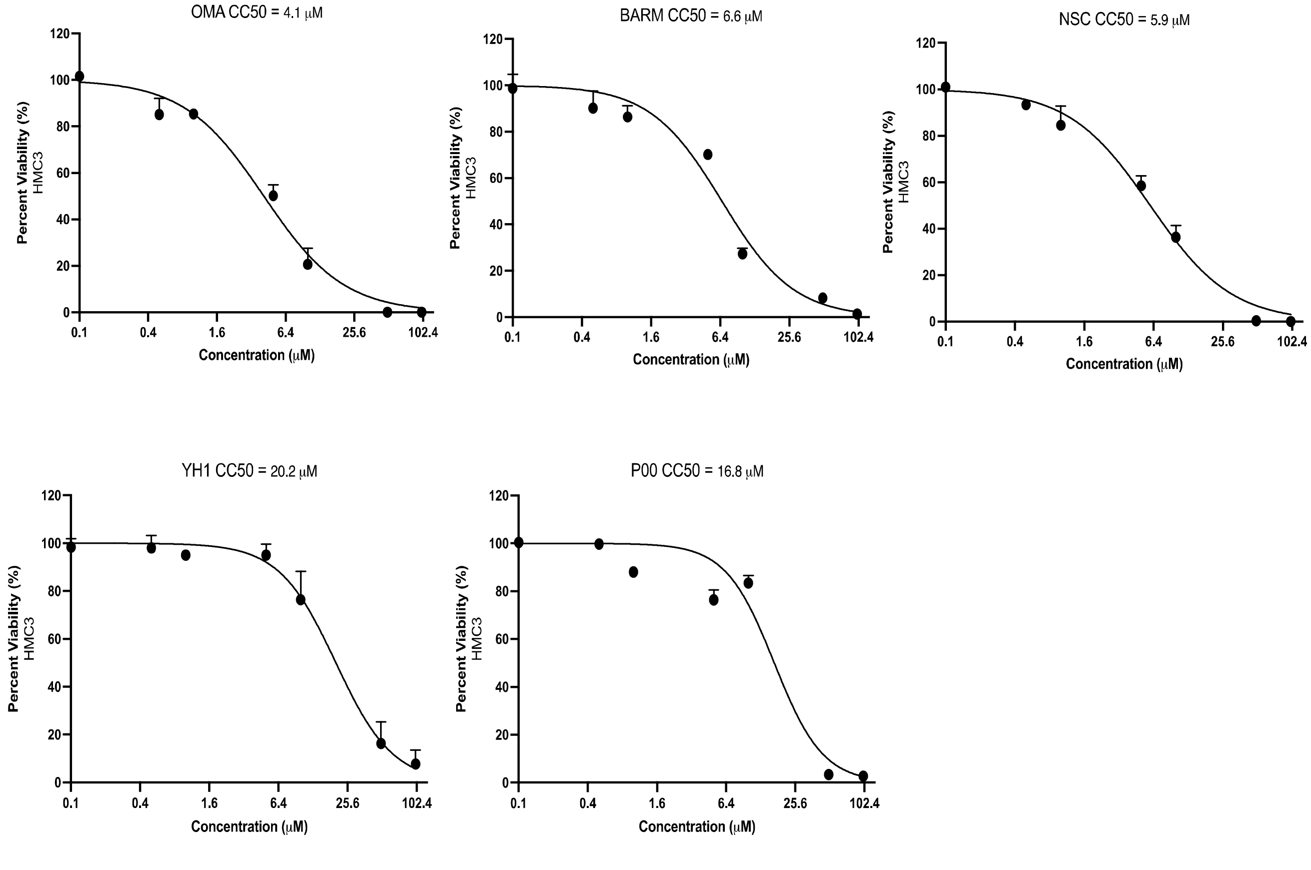

Figure S2. Inhibitors of UPS-mediated signaling inhibit VEEV-TC83 in infected microglial (HMC3) and astroglial (SVG-p12) cells.

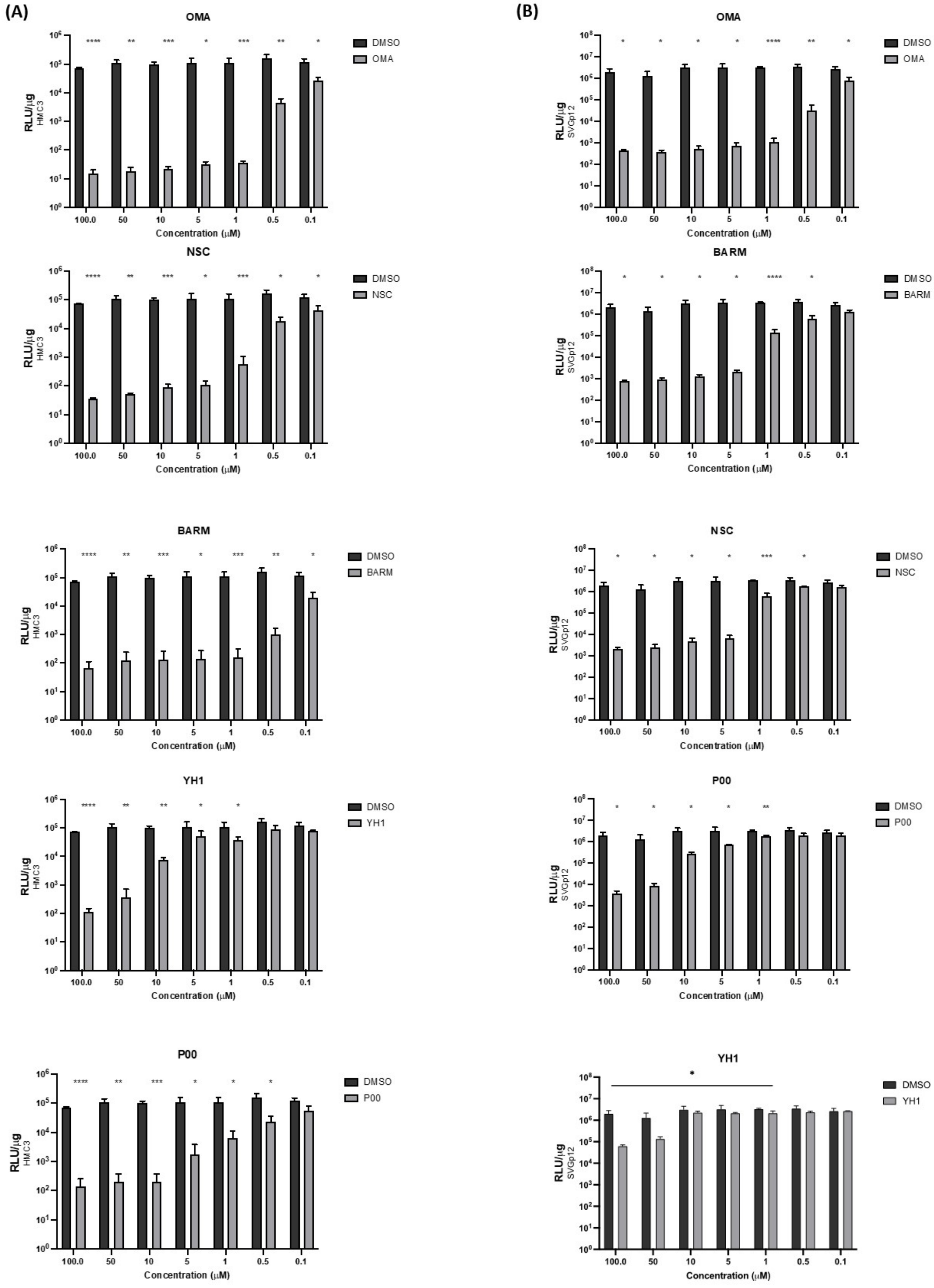

Figure S3. OMA, BARM, and NSC demonstrate inhibition of VEEV-TC83 infectious titer at Moi of 1.

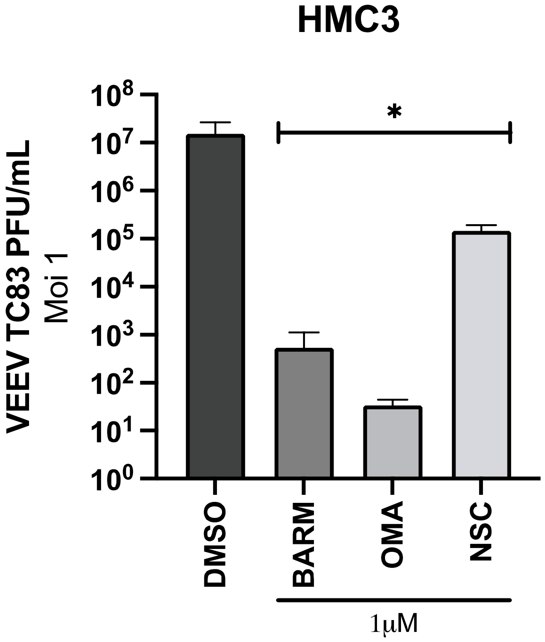

Figure S4. Effect of OMA, BARM, and NSC treatment on expression of proinflammatory cytokines in the supernatants of VEEV-TC83-infected HMC3 cells.

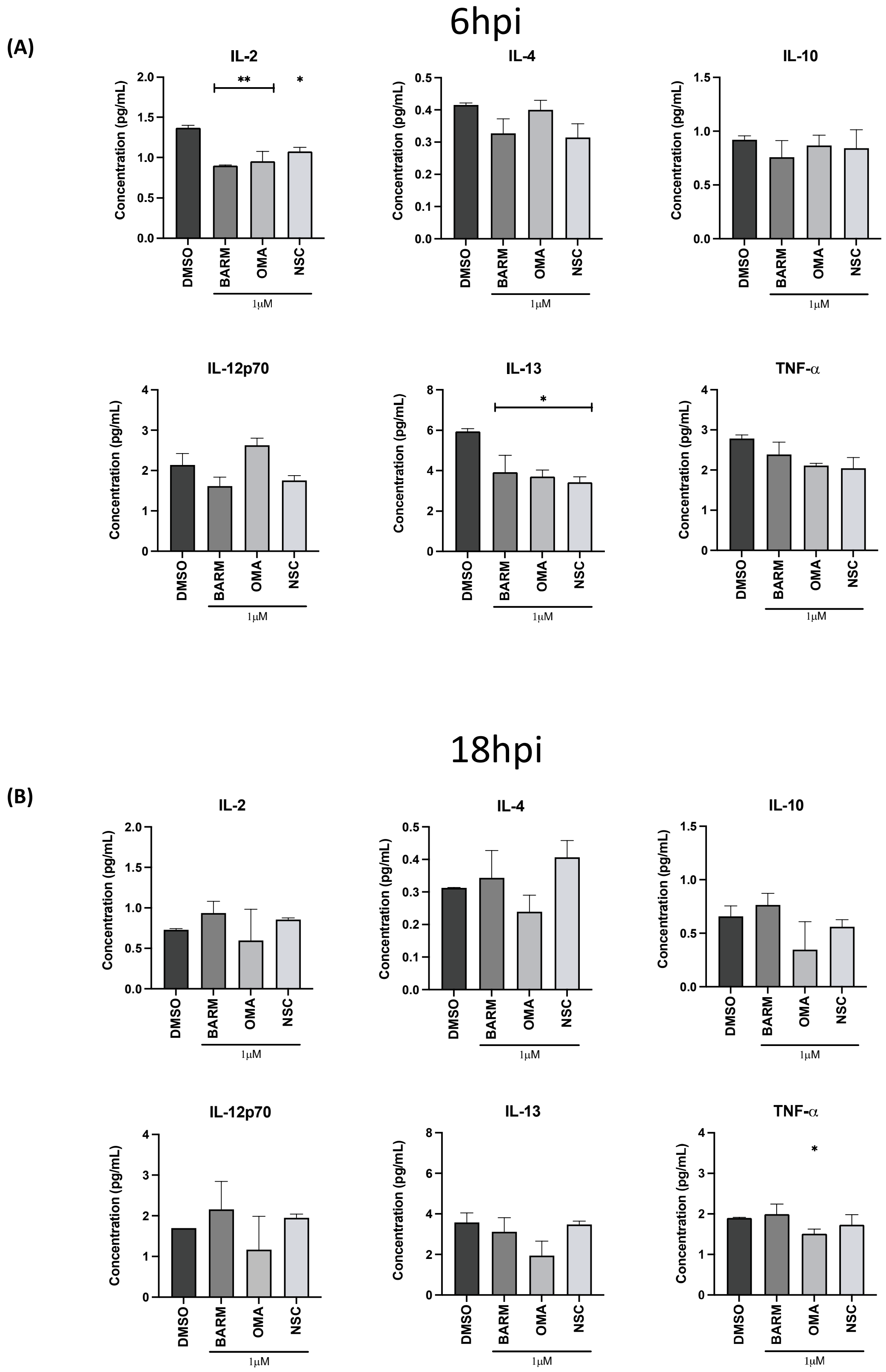

Supplement: Supplementary file 1 [file viruses-15-00655-s001.zip › viruses-2070610-supplementary.pdf]
